# Targeting S1P receptors in experimental autoimmune encephalomyelitis in mice improves early deficits in locomotor activity and increases ultrasonic vocalisations

**DOI:** 10.1038/srep05051

**Published:** 2014-05-23

**Authors:** Graham K. Sheridan, Kumlesh K. Dev

**Affiliations:** 1 Drug Development, School of Medicine, Trinity College Dublin, Dublin, Ireland; 2 Current address: Department of Physiology, Development and Neuroscience, University of Cambridge, Cambridge CB2 3DY, UK.

## Abstract

Fingolimod (FTY720) is an oral therapy for relapsing remitting multiple sclerosis (MS) and targets sphingosine 1-phosphate receptors (S1PRs). FTY720 also rescues animals from experimental autoimmune encephalomyelitis (EAE), an animal model of MS. The protective effects of FTY720 in EAE are primarily scored manually by examining weight loss and limb paralysis that begins around 10–12 days after immunisation. To our knowledge, pre-clinical effects of FTY720 on animal behaviour early in EAE have not been explored. Here, we developed an automated behaviour monitoring system to examine the early effects of FTY720 on subtle pre-symptomatic behaviour of mice induced with EAE. Our automated home-cage monitoring system (AHC-MS) enabled non-contact detection of movement and ultrasonic vocalisations (USVs) of mice induced with EAE, thus allowing detection of subtle changes in mouse behaviour before paralysis occurs. Mice receiving FTY720 emit longer USVs and display higher levels of motor activity than vehicle-treated EAE mice before clinical symptoms become apparent. Importantly, this study promotes the 3Rs ethics (replacement, reduction and refinement) in the EAE animal model and may also improve pre-screening of potentially novel MS therapies. In addition, this is the first report showing the early effects of FTY720 in EAE which underscores its protective effects.

The family of sphingosine 1-phosphate receptors (S1PRs) is G-protein coupled and comprises the subtypes S1P1–5R^1^,^2^,^3^. The oral therapy for relapsing-remitting multiple sclerosis (MS), called fingolimod (FTY720), regulates S1PRs making these receptors *bona fide* drug targets for this illness^4^,^5^. S1PRs are expressed in a number of cell types such as those of the immune and central nervous systems^2^,^3^. The phosphorylated version of FTY720 (pFTY720) works by internalising the S1P1R subtype in T cells, limiting their cellular migration along S1P gradients, egress from lymphoid organs and thus entry into the CNS^6^,^7^,^8^,^9^,^10^. S1P1Rs on endothelial cells are also thought to tighten the blood brain barrier (BBB), in response to pFTY720, likely further restricting T cell movement into the CNS^11^,^12^. Moreover, pFTY720 regulates cytokine release and antibody production from immune cells, which likely contributes to its protective effects in MS^13^,^14^,^15^. Importantly, lipophilic FTY720 readily crosses the BBB where it is phosphorylated to pFTY720 and activates S1PRs expressed in neuronal and glial cells^16^,^17^,^18^. Mounting evidence suggests that pFTY720 can directly alter S1PRs function in these brain cells, which may also be part of this drug's mechanism-of-action in MS^2^,^19^,^20^,^21^,^22^,^23^,^24^.

The animal model that is considered to most closely model multiple sclerosis is termed experimental autoimmune encephalomyelitis (EAE)^25^,^26^,^27^,^28^. EAE is thought to, in some aspects, replicate MS in terms of the inflammation and ensuing CNS demyelination^29^,^30^. This animal model is induced by delivering an antigenic myelin-derived peptide along with an adjuvant that together initiate an inflammatory response producing autoreactive lymphocytes that target the host's endogenous myelin^31^. Usually, over the course of 10–12 days, the animal's immune system attacks myelin and the subsequent motor deficits can be quantified by a standardised manual scoring system that rates the severity of the disease at various stages: no disease (score 0), complete limp tail (score 1), mild, moderate and complete hind limb paralysis (scores 2, 3 and 4, respectively), front limb paralysis and incontinence (score 5), and finally a moribund state (score 6)^32^. While this scoring system is commonly used, those in the field generally note that inherent variability between researchers can occur using this approach^33^. Daily handling can also affect experimental results^34^, for example where stress can raise corticosterone levels in animals, which can alter EAE disease progression^35^,^36^,^37^,^38^. Furthermore, using this clinical scoring system, animals generally reach high disease scores before the beneficial effects of therapies can be assessed^39^,^40^.

Studies show that prophylactic administration of FTY720 reduces the severity of EAE if given from day 0 of EAE induction and also improves motor symptoms if therapeutically administered at the peak of EAE pathology^21^,^41^,^42^,^43^,^44^,^45^,^46^,^47^. A major advantage of FTY720 is that it can be administered orally^48^,^49^ thus minimising the need of daily injections or stressful gavage regimens. In this study, we describe an automated home-cage monitoring system (AHC-MS) that can be used to track locomotion and record ultrasonic vocalisations (USVs) of mice in their home-cage environment. This system was used to detect, in an automated non-contact manner, subtle motor and behavioural abnormalities in EAE mice before the onset of clinical symptoms. We also assess the effects of FTY720 on pre-symptomatic motor and behavioural parameters in EAE mice to validate this model system and further examine the early effects of FTY720 in EAE.

## Results

### FTY720-treated EAE mice show preference for active behaviours compared to vehicle controls

**Figure 1:**
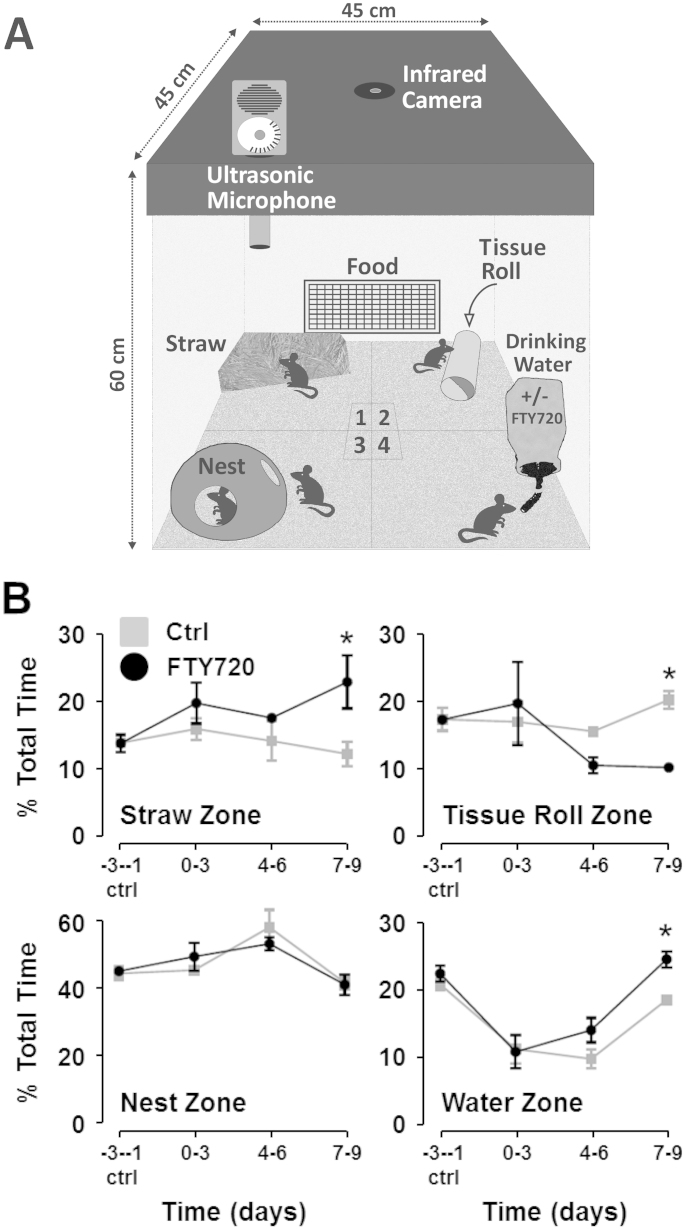
General activity behaviours in EAE mice treated with or without FTY720. (A) Automated Home Cage Monitoring System (AHC-MS). PhenoTyper® home-cages (Noldus) were fitted with top-view camera and infrared LED illumination, Ethovision™ XT tracking software (Noldus) and USV ‘bat recorder' microphone. The Ethovision™ XT tracking software allowed for the demarcation of the home-cage into 4 distinct quadrants. Quadrant 1 contained straw which the mice used to burrow and dig in. Quadrant 2 contained a tissue roll which served as an object the animals could explore and hide in. Quadrant 3 contained the nest which the animals used to sleep in and huddled close together to stay warm. Quadrant 4 contained the drinking water tap supplemented with or without 2 μg/ml FTY720, which the mice received from day 0, i.e. day of EAE induction. Home-cages housed 5 mice induced with EAE. They received *ad libitum* access to food and water. The image/diagram was created by the co-authors (GKS, KKD). (B) Total time spent in each quadrant. The percentage of time the mice spent in each quadrant was calculated overnight (16 hours) between 18:00h–10:00h. The average of days -3 to -1 (i.e. before MOG_35-55_ peptide + CFA subcutaneous injection) was taken as the control baseline and was almost identical for both groups of mice. The averages of days 0–3 and days 4–6 showed no statistically significant differences between control EAE and EAE + FTY720 treated mice. At days 7–9, however, there was a statistically significant difference in the percentage of time spent in quadrant 4 by mice who received FTY720 in their drinking water. EAE mice receiving FTY720 also showed a higher preference for quadrant 1, and a lower preference for quadrant 2 compared to EAE mice not receiving FTY720. This indicates a preference for active behaviours, such as burrowing and digging, over more passive behaviours, such as exploring and hiding, in mice receiving the anti-inflammatory drug, FTY720. * = statistical significance based on a multiple comparisons Student's t test using the Holm-Sidak correction method with an alpha value of 0.05.

In order to investigate the general behaviour of mice induced with EAE, the percentage of time spent in each quadrant of the home-cage (Figure 1A) was calculated over a three day baseline period prior to induction of EAE (days -3 to -1) (Figure 1B). There was remarkable consistency in the times spent in each quadrant between the two groups of mice during this baseline period (Figure 1B). Control EAE mice spent 45.4 ± 2.1% of their time in quadrant 3 (Nest Zone) prior to EAE induction, where they presumably spent a large proportion of that time asleep, compared to 46.1 ± 0.3% for FTY720-treated mice. The duration spent in the Nest Zone did not vary significantly between vehicle control and FTY720-treated mice over the course of EAE disease progression (Figure 1B, *Nest Zone*). The vehicle control EAE group did, however, spend significantly longer in the Nest Zone on days 4–6 post-EAE induction (59.3 ± 5.3%) compared to their baseline period (two-way ANOVA; p value < 0.05) (Figure 1B, *Nest Zone*). There was also a dramatic decrease in the percentage of time spent in quadrant 4 (Water Zone) in both vehicle control and FTY720-treated groups post-EAE induction (20.9 ± 0.9% vs. 11.2 ± 2.1% and 22.5 ± 1.2% vs. 10.8 ± 2.4%, respectively; two-way ANOVA; p value < 0.05) (Figure 1B, *Water Zone*). The time spent in the Water Zone recovered toward baseline levels for both vehicle control and FTY720-treated EAE mice on days 7–9, with the FTY720 treated mice showing a significantly better recovery than vehicle control animals (Figure 1B, *Water Zone*). On days 7–9 post-EAE induction there was also a statistically significant difference in the amount of time each group spent in quadrant 1 (Straw Zone) between vehicle control and FTY720-treated EAE animals (12.3 ± 1.8% vs. 23.0 ± 3.9%) (Figure 1B, *Straw Zone*) and quadrant 2 (Tissue Roll) (20.5 ± 1.4% vs. 10.3 ± 0.1%) (Figure 1B, *Tissue Roll Zone*). The mice in quadrant 2 were generally found hiding inside the tissue roll and thus engaged in relatively passive behaviours (Figure 1B, *Tissue Roll Zone*). In contrast, the mice in quadrant 1 were foraging, burrowing and gathering nesting material thus displaying more active behaviours. Therefore, compared to vehicle control, the mice receiving FTY720 appeared generally more active suggesting early beneficial effects of FTY720 in EAE.

### FTY720 treatment improves locomotor activity in mice at early EAE stages

**Figure 2:**
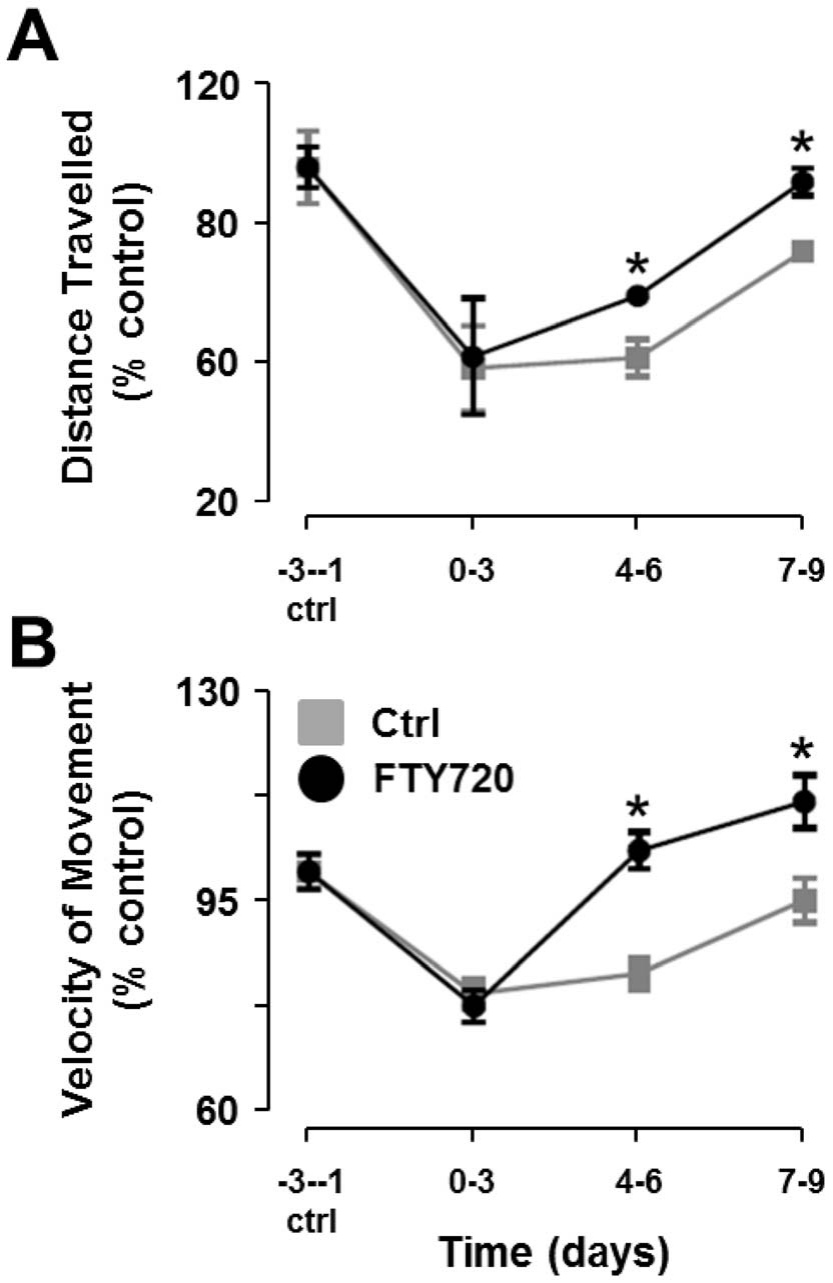
Locomotor activity in control EAE mice treated with or without FTY720. (A) Distances travelled. The average distances travelled by the mice each day were calculated and the baseline was taken as the average of days -3 to -1. This value was set to 100% for each group of mice and the subsequent deviations in distance travelled post-EAE induction were calculated as a percentage of the baseline. The locomotor activity of the mice drastically reduced over the first 4 days after EAE induction in all mice. The mice treated with FTY720 showed a significantly quicker recovery time toward baseline values over the subsequent 6 days compared to the mice that received no drug. (B) Velocities of movements. The baseline was set to 100% and was calculated based on the average of the velocity of movement 3 days before EAE induction (days -3 to -1). There was a significant reduction in the velocity of movement over the first 4 days post-EAE induction in all mice. The mice treated with FTY720 recovered their locomotion speed quicker than control EAE mice over the subsequent 6 days. * = statistical significance based on a multiple comparisons Student's t test using the Holm-Sidak correction method with an alpha value of 0.05.

In addition to the altered times spent in the four quadrants between vehicle control and FTY720-treated EAE mice, we also found a reduction in the distance travelled by EAE mice in the first 4 days post-EAE induction for both vehicle control (52.0 ± 10.2%) and FTY720-treated (54.7 ± 14.0%) groups (Figure 2A). Likewise, the velocity at which the mice moved in these first 4 days was also reduced for vehicle control (79.5 ± 0.8%) and FTY720-treated (77.5 ± 0.8%) groups (Figure 2B). This significant reduction in activity 4 days post-EAE induction is likely due to a moderate amount of pain, irritation and anxiety caused by the sub-cutaneous and intra-peritoneal injections of MOG_35–55_/CFA and pertussis toxin. Notably, mice treated with FTY720 made a faster recovery and, on days 4–9 post-EAE induction, showed statistically significant increases in the distances travelled (54.5 ± 4.4% vs. 69.2 ± 0.8% on days 4–6 and 79.8 ± 1.7% vs. 96.5 ± 3.3% on days 7–9) (Figure 2A) and their speed of movement (82.9 ± 0.6% vs. 103.6 ± 0.7% on days 4–6 and 95.2 ± 0.9% vs. 111.9 ± 1.0% on days 7–9) (Figure 2B) around the cage compared to vehicle control counterparts.

### FTY720-treated EAE mice emit longer USVs than vehicle control EAE mice

**Figure 3:**
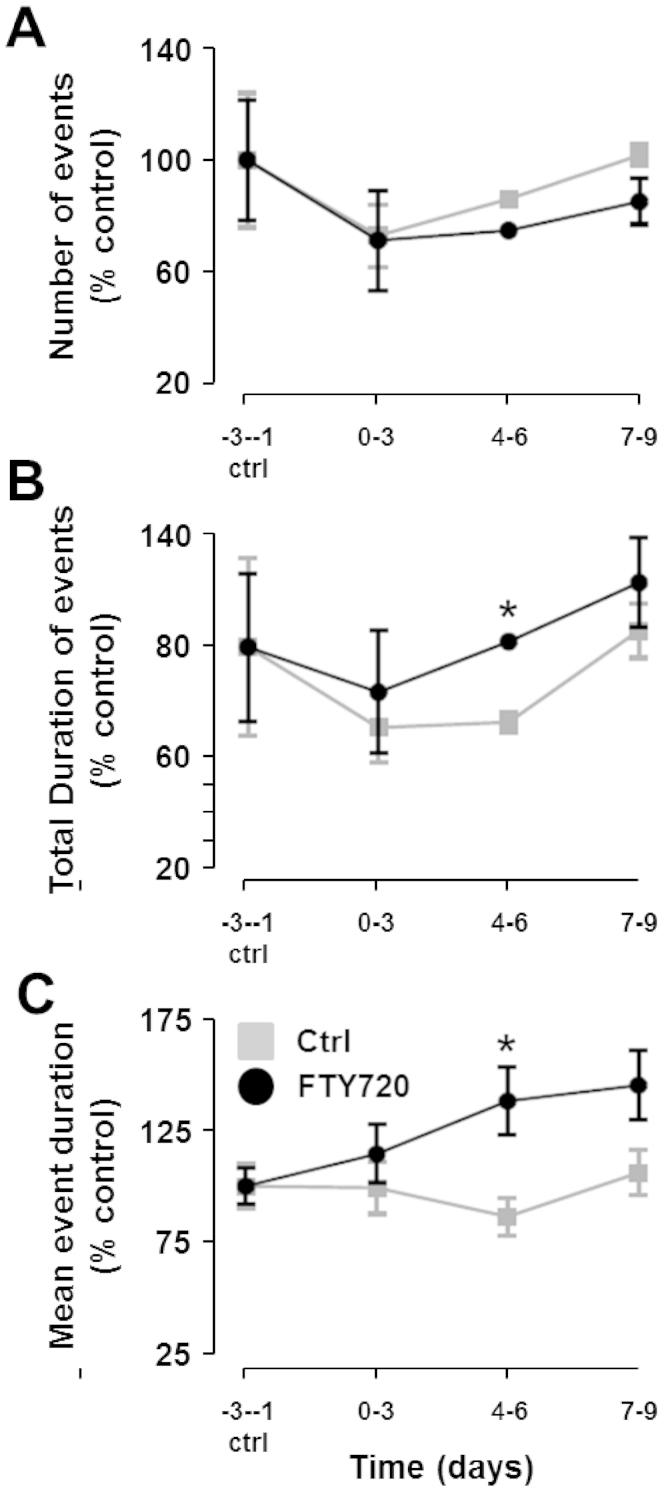
USVs in control EAE and EAE + FTY720 treated mice. (A) Number of USVs (50 kHz), per hour. The baseline number of USV events was set at 100% for each group of mice and calculated as the average of the total number of events each day, three days before EAE induction (days -3 to -1). There were no significant changes over the course of EAE progression in the number of USVs from either control EAE or EAE + FTY720 treated mice. (B) Total Duration of USVs (50 kHz). The total duration the mice spent emitting USVs decreased significantly in control EAE mice post-EAE induction but returned to baseline levels 7–9 days post-immunisation. There was a statistically significant difference in the total duration of USVs between control and FTY720-treated mice on days 4–6 post-EAE induction. (C) Mean Duration of each 50 kHz USV. The mean duration of each individual USV event did not vary significantly from the baseline in control EAE mice. Mice induced with EAE and treated with FTY720, however, showed a statistically significant increase in the mean duration of each USV event on days 4–6 and days 7–9 post-EAE. The mean duration of each event also differed significantly from control EAE mice on days 4–6 post-EAE. * = statistical significance based on a multiple comparisons Student's t test using the Holm-Sidak correction method with an alpha value of 0.05.

We next measured collective USV events (50 kHz) emitted by each group of mice over the 12 day time-course of the experiment. There was no statistically significant change from baseline in the number of USVs emitted by either vehicle control or FTY720-treated group post-EAE induction (Figure 3A). Likewise, there were no significant differences in the number of events between vehicle control and FTY720-treated EAE mice (Figure 3A). The total duration of USV events was, however, significantly longer in FTY720-treated EAE mice compared to vehicle control EAE mice on days 4–6 post-EAE induction (Figure 3B). This difference was due to an increase in the mean duration of each USV event in FTY720-treated mice (138.5 ± 15.3% of baseline duration) (Figure 3C). The mean duration of each USV event increased even further to 145.6 ± 15.7% of the baseline on days 7–9 post-EAE induction in FTY720-treated mice (Figure 3C). In contrast, the mean duration of USV events remained relatively constant in control EAE mice over the course of the disease (Figure 3C). This suggests, therefore, that EAE does not alter mean 50 kHz USV duration. FTY720 treatment, on the other hand, increases mean USV duration. Whether this is an indication of increased social interaction in FTY720-treated mice compared to drug-free controls remains to be confirmed.

## Discussion

At present, pre-clinical evaluation of potential therapeutics for MS is generally determined by investigating their effectiveness in preventing/treating EAE, an animal model of MS. The symptomatology in EAE requires 10–12 days to manifest and the motor impairments are quantified manually using a standardised scoring system. Our aim, in this study, was to develop an automated system with the ability to detect pre-symptomatic abnormalities in locomotion and behaviour in mice before the overt motor disabilities of EAE emerged. To achieve this, we used a well characterised protocol where C57BL/6 mice were induced with a chronic progressive form of EAE using the MOG_35–55_ peptide as the antigen^50^,^51^. We also developed an automated home-cage monitoring system (AHC-MS) that was able to detect subtle motor impairments in mice earlier than a manual scoring method. This AHC-MS had the following notable features: 1) it allowed visual recording and tracking, in real-time, of animal locomotion in their home-cage environment, 2) it had a large enriched environment to promote more natural animal behaviours such as exploration, foraging, burrowing and nest-building activities, which could also be monitored remotely, 3) it minimised handling of animals, thus potentially reducing anxiety and stress, 4) it allowed animals to be monitored for long periods overnight when they are at their most active, 5) it recorded collective USVs of mice, and 6) it minimised unintentional experimenter bias via automated recordings.

In order to validate this AHC-MS as an effective screening platform for MS therapeutics, we assessed the effects of the oral drug, FTY720, on the early stages of EAE in mice. All mice were monitored for 10 days post-immunisation with MOG_35–55_ and maintained a clinical score of 0 for the duration of the experiment. Pre-symptomatic abnormalities in locomotion and USVs were analysed in vehicle- versus FTY720-treated EAE mice. Our results confirmed the protective effects of FTY720 on EAE-induced motor deficits and demonstrated beneficial effects of the drug up to one week prior to clinical symptomatology onset; far sooner than previous studies have detected. We found, compared to vehicle-treated EAE mice, the FTY720-treated EAE mice displayed a preference for active behaviours including foraging, burrowing and gathering nesting material. Drug-treated mice also displayed greater locomotor activities, namely, distance travelled and speed of movement. Interestingly, EAE induction did not alter the number or the duration of group USVs emitted during the early disease period. However, FTY720-treated EAE mice emitted USVs of longer duration on days 4–6 post-EAE induction. This increase in USV duration coincided with an increase in locomotor activity in FTY720-treated EAE mice and may also correspond to an increase in social interaction, since FTY720-treated mice spent more time burrowing, exploring and nest-building 7–9 days post-EAE induction. These vocalisations appeared to also associate with increased activity in mice since they directly correlated with a return-to-baseline of normal velocity and distance travelled post-EAE.

At this stage, we cannot rule out a direct or indirect CNS-based pharmacological mechanism underlying the increased USV duration recorded in FTY720-treated mice. It has previously been shown that some pharmacological treatments can alter USVs in rodents. For example, morphine has been demonstrated to reduce 50 kHz USVs^52^, whereas the GABA_A_ receptor antagonist, pentylenetetrazol, can cause an increase in USVs in rats^53^. Therefore, FTY720 may increase 50 kHz USVs in mice through pharmacological actions on S1PRs in the CNS and/or in other systems where S1PRs are expressed. In addition, it is worth noting that environmental enrichment has been observed to increase USVs in rodents^54^ and that emission of 50 kHz vocalisations correlates with positive affect in rats^55^,^56^, although their function in mice is less clearly defined^57^. Moreover, vocal communications between female mice in the 50–70 kHz frequency range have been positively correlated with various social interaction situations^58^. It is tempting to attribute FTY720's increase in mean USV event duration described here with a more positive emotional state and increased social communication among group-housed female mice. However, interpreting the physiological and emotional meaning of USVs in mice, in particular, is still controversial.

Previous studies have shown that behavioural and emotional stress in rodents can sometimes inhibit or accelerate EAE disease progression, depending on the timing and duration of the stressor^35^,^36^,^37^,^38^; where, for example, over-activation of the hypothalamic-pituitary-axis (HPA) through raised corticosterone levels can suppress immune function. The administration of FTY720 to animals in drinking water results in similar therapeutic responses in EAE compared to daily i.p. injections of drug^48^,^49^. We thus took advantage of FTY720's capacity to be administered via the drinking water and used it, at a similar concentration to that suggested by previous studies, in our AHC-MS^48^,^49^. We therefore avoided daily intra-peritoneal injections or oral gavage allowing reduced handling and stress associated with these drug administration methods. Notably, there was no difference in the volume of water consumed per day by vehicle- and FTY720-treated groups of EAE mice (approx. 15 ml/group/day) over the course of the disease.

Overall, this study is a proof-of-principle that the developed AHC-MS is sensitive to early pre-symptomatic motor deficits in mice with EAE. It also demonstrates the early protective effects of FTY720. While it will be necessary to assess the effects of other anti-inflammatory compounds, also known to be beneficial in EAE, this system has the potential to improve experimental methods associated with manual clinical scoring. We do not see this AHC-MS as a complete replacement to the current clinical scoring methods, but rather an additional tool that can be used alongside gold standard routines to assess beneficial drug responses in EAE. The most sensitive parameter describing FTY720's therapeutic effects in the early phase of EAE was the return-to-baseline in the animal's velocity of movement on days 4–6 post-immunisation. It would be important when testing other drugs for potential therapeutic benefit in EAE, to assess if they also exert similar improvements in animal locomotion at these early stages post-EAE induction. An important advantage of this AHC-MS is its versatility. The home-cage can be easily modified to detect alterations in different types of animal behaviour. For example, novel and familiar objects could be placed into the home-cage each day to assess the effects of drugs on memory impairment or reduced motivation associated with EAE^59^. Moreover, the effects of drugs on more complex motor tasks such as wheel-running or beam-walking onto elevated platforms could easily be implemented using this system. An obvious disadvantage of our AHC-MS is the initial cost involved in purchasing large automated home-cages; although it is certainly feasible to build custom-designed home-cages using relatively inexpensive materials and readily affordable video and audio recording technology. Moreover, the space restrictions within standard animal facilities may restrict the number of home-cages that can be monitored in parallel. Another limitation of this method is that most pre-clinical compounds do not have as good oral bioavailability as FTY720 and cannot be administered to mice through their drinking water. Therefore, manual restraint and injection of test compounds may be necessary in certain situations. The confounding anxiogenic effects of these drug-delivery methods could be minimised, however, by injecting the mice first thing each morning after overnight video tracking is complete.

Finally, we believe this AHC-MS would also provide a means to better satisfy the 3Rs ethical policy in animal research, i.e. replacement, reduction and refinement^60^. Examining the effects of novel compounds at these early time points in EAE should also shorten experimental time-frames and may improve and broaden our mechanistic understanding of therapies used in MS.

## Methods

### Animal Care and Automated Home Cage Monitoring System (AHC-MS)

Postnatal day 60 C57BL/6 female mice (25–30 g) were obtained from Harlan and housed in the Bioresources Unit, Trinity College Dublin, Ireland. All experimental procedures were approved by the animal research ethics committee and head veterinarian of the Bioresources Unit, TCD and were carried out by individuals who held the appropriate license issued by the minister for health and children. The methods were carried out in accordance with the approved guidelines. Animals were socially housed in groups of 5 in standard laboratory cages, allowed to acclimatise to the new facility for 5 days and given *ad libitum* access to food and water. The experimental room was kept on a 12 h light/dark cycle at 22 ± 2°C. Mice were then transferred, in the same social groups, to the automated home-cage monitoring system (AHC-MS), i.e. the commercially available PhenoTyper® home-cage (Noldus, Wageningen, Netherlands) (Figure 1A). The mice were allowed to acclimatise to these larger enriched environment cages for a further 5 days. The cage was demarcated into four distinct quadrants. Quadrant 1 contained a large bundle of straw which was added to enable the mice to enact their natural propensity to burrow, dig and nest-build. A standard tissue roll was placed in Quadrant 2 which, in the beginning, served as a novel object for the mice to explore but, after some time, the animals used it to climb on and hide in. Quadrant 3 contained a red opaque plastic nest which the animals used to sleep in and huddle close together to stay warm. Quadrant 4 contained the drinking-water tap which contained water either unsupplemented (i.e. control EAE group) or supplemented with 2 μg/ml FTY720 (i.e. EAE + FTY720 group). Normal rodent chow was accessible through a large grill located close to the floor of the cage and spanned quadrants 3 and 4. The roof of the PhenoTyper® cage was approx. 60 cm from the cage floor (45 × 45 cm) and was fitted with infrared LED lights and a wide field of view camera connected to an external computer running Ethovision™ XT software (Noldus). The roof of the cage also housed an ultrasound detector (Mini-3 bat model, connected to Ultravox software running on an external PC) which was used to detect USVs in a specific frequency range. We tested several frequency ranges from 40–80 kHz over the five day acclimatising period and found USVs detected in the 50 kHz range delivered the largest and most consistent number of events between cages. Since the bat recorder only allowed us to record within a narrow frequency range, we chose 50 kHz as the optimal frequency to detect throughout the whole experiment. After 5 days, the bedding of the cages was changed and fresh food and water were added. Baseline measurements of locomotor activity and USVs were then recorded for 3 days prior to EAE induction (i.e. days -3, -2, -1). Recordings were conducted for 16 hours each day overnight from 18:00h to 10:00h. This was to ensure that no other researchers or animal technicians would enter the room and disturb the mice or recordings. Since the mice are nocturnal and mostly sleep during the day, this allowed us to record them at their peak daily activity; which remains a limiting paradigm for manual EAE scoring. The locomotion (i.e. speed and distance travelled) was averaged each day and an average of the first 3 days was taken as the ‘baseline' for each group of animals. These baseline values were set to 100%. Average daily variations in locomotor activity post-EAE induction for each group of mice were compared to these baseline values.

### MOG_35 – 55_ peptide EAE induction protocol and FTY720 administration

Mice were immunised by sub-cutaneous injection (s.c.) of 100 mg MOG_35–55_ peptide (GenScript) emulsified in Complete Freund's Adjuvant (CFA) containing 4 mg/ml (0.4 mg/mouse) of heat-inactivated M. tuberculosis (Chondrex). Mice were also injected intra-peritoneal (i.p.) with 500 ng pertussis toxin (Kaketsuken) on days 0 and 2. This method has been shown to consistently induce a chronic progressive form of EAE in 100% of immunised mice. This chronic EAE consists of a single peak in symptomatology beginning around days 10–12 and shows maximal penetrance around days 17–22 followed by a gradual (but incomplete) improvement in motor symptoms from day 25 onwards^61^. In this study, animals were humanely euthanized prior to motor deficit onset on day 9 post-EAE induction. In this way, we aimed to develop a sensitive, automated and non-contact method for detecting early motor impairments in EAE as well as devising an improved protocol for screening novel drugs with the added advantage of reducing the numbers and suffering of laboratory animals by implementing the 3Rs policy. Two groups of 5 mice were used in this study, i.e. Control EAE and EAE + FTY720. The EAE + FTY720 group of mice received *ad libitum* access to drinking water containing 2 μg/ml FTY720. The Control EAE group received normal drinking water.

### Data collection and statistical analysis

Locomotor activity was recorded directly from the camera in the ceiling of the cage to an external PC using Ethovision™ XT software (Noldus). USV events in the 50 kHz frequency band were recorded using a bat detector microphone (Mini-3 bat model) and processed in real-time by Ultravox software which saved the data onto the same external PC as 1) number of USV events, 2) total duration of events and 3) mean USV event duration. Recordings were acquired each day for 16 hours between 18:00h and 10:00h overnight. Data was collected in Microsoft Excel and imported into GraphPad Prism statistical software. Data was analysed using either 1) multiple comparisons Student's t test using the Holm-Sidak correction method with an alpha value of 0.05; or 2) two-way ANOVA with Tukey correction for multiple comparisons and p value < 0.05 indicating significance.

## Author Contributions

G.K.S. and K.K.D. designed the study. G.K.S. performed experiments and analysed the data. G.K.S. and K.K.D. wrote the paper and prepared the figures.
